# Investigation of BECN1-Mediated Autophagy Mechanisms Triggered by External Stimuli in Clinical Mastitis of Dairy Cows

**DOI:** 10.3390/biom16010133

**Published:** 2026-01-12

**Authors:** Nong Cai, Bohao Zhang, Na Chen, Jiayu Yue, Jianfu Li, Weitao Dong, Yong Zhang, Xingxu Zhao, Quanwei Zhang

**Affiliations:** 1 College of Life Science and Technology, Gansu Agriculture University, Lanzhou 730070, China; 1073323020308@st.gsau.edu.cn (N.C.); zhangbh@st.gsau.edu.cn (B.Z.); 1073323020311@st.gsau.edu.cn (N.C.); zhychy@163.com (Y.Z.); zhaoxx@gsau.edu.cn (X.Z.); 2 Gansu Key Laboratory of Animal Generational Physiology and Reproductive Regulation, Lanzhou 730070, China; yuejiayu@gsau.edu.cn (J.Y.); 1073325140048@st.gsau.edu.cn (J.L.); dongwt@gsau.edu.cn (W.D.); 3 College of Veterinary Medicine, Gansu Agriculture University, Lanzhou 730070, China

**Keywords:** clinical mastitis, external stimuli, BECN1, autophagy

## Abstract

Disruption of the blood–milk barrier and inhibition of enzymatic activity caused by abnormal external stimuli, accompanied by the occurrence of autophagy, are among the major factors contributing to the onset of clinical mastitis (CM) in dairy cows. However, the molecular mechanisms through which external stimuli and autophagy regulate CM in dairy cows are not fully understood. This study examined mammary gland (MG) tissue samples collected from healthy dairy cows and those with CM caused by *Staphylococcus aureus* (*n* = 3 per group) to observe histological changes and autophagic phenomena, identify candidate biomolecular targets involved in external stimuli in dairy cows affected by mastitis through proteomic and bioinformatic analyses, and analyze their expression and distribution patterns in MG tissues. Pathological examination revealed that the MG tissues of the CM group exhibited significant alveoli collapse and inflammatory cell infiltration, accompanied by autolysosome and phagolysosome activation, and elevated expression of lysosomal and autophagic markers. Bioinformatic analysis identified five biological processes (BPs) and 144 differentially expressed proteins (DEPs) associated with external stimuli, among which beclin 1 (BECN1) was involved in all five BPs. Pathway enrichment analysis revealed that BECN1 participated in six autophagy-related signaling pathways. BECN1 was localized in the cytoplasm of mammary epithelial cells, and both mRNA and protein levels of *BECN1* were significantly upregulated in the CM group compared with those in the controls (*p* < 0.01). These findings suggest that BECN1 expression is closely associated with CM in dairy cows and correlates with autophagy-related responses to external stimuli, and its elevated expression is positively correlated with *Staphylococcus aureus*–induced CM severity. Our results offer preliminary observations relevant to the molecular mechanisms by which BECN1, the autophagy-regulating biomolecule BECN1 influences the development of CM.

## 1. Introduction

Bovine mastitis is one of the most common diseases in the dairy industry and severely affects the cow’s health, milk production, and milk quality [1]. Clinical mastitis (CM), a subtype of bovine mastitis, poses a serious threat to dairy cow health [2,3]. CM is closely associated with external stimuli such as microbial infections and mechanical injuries. Various adverse external factors can disrupt the integrity of the mammary gland (MG) tissue barrier and interfere with cellular metabolism, and activate autophagy. However, the molecular mechanisms by which external stimuli and autophagy regulate CM in dairy cows remain unclear.

Microbial infections (such as *Staphylococcus aureus* and *Escherichia coli*), mechanical injuries, oxidative stress, and exposure to environmental toxins are the major categories of external stimuli implicated in the induction of CM in dairy cows. Although these stimuli differ with regard to their origin, they elicit overlapping host cellular response programs that can be systematically captured by biological processes related to the responses to external or extracellular stimuli. Although these stimuli differ in origin, these factors converge on common host cellular response pathways [4,5]. Pathogenic microorganisms are recognized by pattern recognition receptors on mammary epithelial cells (MECs) via pathogen-associated molecular patterns, which trigger inflammatory responses and activate immune signaling pathways [6,7,8]. Mechanical stress during milking can disrupt tight junctions in MECs and exacerbate local inflammation [9]. Persistent or excessive external stimulation disrupts mammary immune homeostasis and is a key inducer of CM [6], and continuous stimulation can cause immune over-activation, oxidative stress, and cellular damage in MECs. For instance, *Staphylococcus aureus*-toxin activates the mitochondrial protease high-temperature requirement protein A2, which promotes the degradation of the anti-apoptotic protein X-linked inhibitor of apoptosis protein and the mitochondrial fusion protein optic atrophy 1, leading to mitochondrial dysfunction, MEC apoptosis, and exacerbation of mastitis pathology [10,11]. Thus, external stimuli may cause mastitis and accelerate its pathological progression.

In addition to inducing inflammatory responses, external stimuli disturb cellular homeostasis by causing mitochondrial dysfunction, increased production of reactive oxygen species, and endoplasmic reticulum (ER) stress. These abnormal signals can activate autophagy, which eliminates damaged organelles and maintains cellular homeostasis [12,13]. Oxidative stress and ER stress induced by bacterial infection can trigger autophagy in MECs as a compensatory defense mechanism [14]. *Staphylococcus aureus* and *Escherichia coli* infections can induce autophagy in MECs, thereby alleviating inflammatory injury [15]. Autophagy can also modulate immune cell (macrophage and T cell) functions, suppressing the excessive release of proinflammatory cytokines such as tumor necrosis factor-α (TNF-α) and interleukin (IL)-6 to mitigate tissue injury caused by mastitis [16]. Thus, autophagy serves as an innate immune defense mechanism that eliminates invading pathogens and damaged cellular components, thereby protecting host cells [17]. However, excessive or dysregulated autophagy can have deleterious effects. For instance, activation of the calmodulin-dependent protein kinase II β/AMP-activated protein kinase (AMPK) signaling pathway in a high-concentrate diet-induced metabolic inflammation model results in excessive autophagy, causing MG tissue injury and inflammatory cell infiltration [18,19]. Thus, autophagy contributes to the maintenance of tissue homeostasis; however, aberrant or excessive autophagy may become a key factor that exacerbates inflammation and tissue damage.

In this study, MG tissues were collected from lactating healthy Holstein cows (control, Con) and cows with CM. Hematoxylin–eosin (H&E) staining, transmission electron microscopy (TEM), immunohistochemistry (IHC), immunofluorescence (IF), quantitative reverse-transcription PCR (qRT-PCR), and Western blot (WB) were performed to examine histological, ultrastructural, and molecular changes. In addition, data-independent acquisition (DIA)-based proteomic analysis was used to screen biological processes (BPs), pathways, and key differentially expressed proteins (DEPs) associated with the keyword “external stimuli.” This study aimed to elucidate the biomolecular targets and potential mechanisms through which abnormal external stimuli and autophagy regulate the occurrence and development of bovine CM, thereby providing a theoretical basis for the development of novel prevention and control strategies of CM in dairy cows.

## 2. Materials and Methods

### 2.1. Sample Preparation and Collection

MG tissue samples were collected from a large-scale dairy farm in Wuzhong City, Ningxia, China. Lactating Holstein cows were selected based on veterinary clinical diagnosis, somatic cell count (SCC), Lanzhou mastitis test (LMT) results, and pathogen isolation and identification, and were categorized as healthy (control group, Con) or CM induced by *Staphylococcus aureus* (*n* = 3 per group) [20]. The selected cows were transported to a local abattoir, where fresh MG tissues were collected immediately after slaughter. Portions of the tissue samples were fixed in 4% paraformaldehyde or 2.5% glutaraldehyde for histological and ultrastructural analyses, and the remaining samples were snap-frozen in liquid nitrogen and stored at −80 °C. All experimental procedures involving animals were reviewed and approved by the Ethics Committee of Gansu Agricultural University (No. GSAU-Eth-VMC-2021-020).

### 2.2. Hematoxylin-Eosin Staining

MG tissues were fixed and processed using standard histological procedures to prepare 5 μm paraffin sections [21]. After baking at 60 °C, the sections were deparaffinized with xylene and rehydrated using a graded ethanol series. H&E staining was performed using a commercial staining kit (ServiceBio, Wuhan, China). Stained sections were observed and imaged using a light microscope equipped with an imaging system (Nikon, Tokyo, Japan).

### 2.3. Transmission Electron Microscopy

The ultrastructural morphology of lysosomes in bovine MG tissues was examined using TEM. As previously described [22], after fixation, rinsing, and dehydration at 25 °C, the samples were embedded in resin and sectioned into ultrathin slices (60−80 nm). The sections were stained using 2% uranyl acetate and 2.6% lead citrate. Ultrastructural features were observed and imaged using a Hitachi HT7800 transmission electron microscope (Hitachi, Minato-ku, Japan).

### 2.4. Immunofluorescence Staining

The paraffin-embedded tissue sections were deparaffinized, rehydrated, and subjected to antigen retrieval. Endogenous peroxidase activity was quenched with 3% H_2_O_2_, and nonspecific binding was blocked with 5% donkey serum (Solarbio, Beijing, China). The sections were incubated overnight at 4 °C with the following rabbit anti primary antibodies: cytokeratin 18 (CK18), BECN1 (1:200; Proteintech, Wuhan, China; Table S5), lysosomal-associated membrane protein 2 (LAMP2), and microtubule-associated protein 1 light chain 3 (LC3) (1:200; ServiceBio; Table S5). Phosphate-buffered saline (PBS) was used instead of the primary antibody as a negative control. Subsequently, the sections were incubated with an Alexa Fluor-conjugated goat anti-rabbit IgG (H&L) secondary antibody at 37 °C. The nuclei were counterstained using 4′,6-diamidino-2-phenylindole (DAPI; Solarbio), and the sections were mounted using an antifade mounting medium (Solarbio). Fluorescence images were captured using a fluorescence microscope (Olympus, Tokyo, Japan).

### 2.5. Bioinformatics Analysis

Bioinformatic analyses were performed using previously obtained DIA proteomic data of MG tissues from Con and CM cows (deposited in the ProteomeXchange repository under accession IPX0003382000/PXD028100), with Gene Ontology (GO) and Kyoto Encyclopedia of Genes and Genomes (KEGG) enrichment analysis conducted using R software packages v4.3.3. BPs were screened using “external stimulus” as a keyword, including terms such as “response to external stimulus” and “response to extracellular stimulus,” to identify core DEPs, and the involvement of these core DEPs in pathways was subsequently evaluated. Data visualization, including enrichment chord diagrams, heat maps, volcano plots, upset diagrams and bubble charts, was performed using the online OmicShare tools (https://www.omicshare.com/tools/, accessed on 20 February 2025). Protein–protein interaction (PPI) networks were constructed using STRING v.12.0 (https://cn.string-db.org/, accessed on 23 February 2025) and further visualized and analyzed using Cytoscape v.3.9.19 (Cytoscape Consortium, La Jolla, CA, USA) with the ClueGO plugin v 2.5.10.

### 2.6. Immunohistochemistry Staining

Paraffin-embedded MG sections were deparaffinized, rehydrated, and subjected to antigen retrieval in a sodium citrate buffer (ServiceBio). Endogenous peroxidase activity was blocked using 3% hydrogen peroxide (H_2_O_2_), followed by blocking using bovine serum albumin (Solarbio). The sections were then incubated overnight at 4 °C with a rabbit anti-BECN1 primary antibody (1:500; Proteintech; Table S5). For the negative control, PBS was used instead of the primary antibody. Secondary antibody and streptavidin–biotin complex were subsequently applied, according to the manufacturer’s instructions [23]. Sections were visualized using 3,3′-diaminobenzidine (Solarbio), counterstained with hematoxylin, dehydrated, cleared, and mounted. Images were captured using a microscope (Nikon), and at least five random fields were selected per section. Image-Pro Plus software (version 7.0; National Institutes of Health, Rockville, MD, USA) was used to analyze the integrated optical density (IOD) of BECN1 staining, with each sample analyzed in triplicate.

### 2.7. RNA Isolation, cDNA Synthesis, and qRT-PCR

Total RNA was extracted from MG tissues using an RNA extraction kit (Vazyme, Nanjing, China) according to the manufacturer’s instructions [23]. One microgram of total RNA was reverse-transcribed into complementary single-stranded DNA (cDNA) using an Evo M-MLV reverse-transcription premix kit (Accurate Biotechnology, Changsha, China). The synthesized cDNA was stored at −80 °C [24]. The relative mRNA expression level of *BECN1* in MG tissues was determined using qRT-PCR, with β-actin as the internal reference gene. qRT-PCR was performed using a LightCycler 96 Real-Time PCR System (Roche, Basel, Switzerland). qRT-PCR primers (Supplementary Table S1) were designed using Primer3 v 4.1.0 (https://primer3.ut.ee/, accessed on 25 March 2025) and synthesized by Qingke Biotech (Xi’an, China). The relative expression levels of target genes were calculated using the 2^−ΔΔCt^ method [25].

### 2.8. Western Blot

Approximately 80 mg of MG tissue was used for total protein extraction. Samples were lysed in radioimmunoprecipitation assay buffer (Solarbio) supplemented with 1% phenylmethylsulfonyl fluoride (Solarbio) and 1 mmol/L protease inhibitor cocktail (Solarbio), as described previously [24,26]. The protein concentrations were determined using a bicinchoninic acid assay kit (Solarbio). Equal amounts of protein (30 μg per sample) were separated by 12% sodium dodecyl sulfate–polyacrylamide gel electrophoresis and transferred onto polyvinylidene fluoride membranes. The membranes were incubated overnight at 4 °C with rabbit anti-BECN1 or β-actin primary antibodies (1:4500; Proteintech; Table S5). Then, the membranes were incubated with horseradish peroxidase–conjugated secondary antibody (1:5000; Bioss, Beijing, China; Table S5) for 1 h at 37 °C. Protein bands were visualized using enhanced chemiluminescence (NCM, Suzhou, China) reagents. The intensity of each target protein band was measured using Image-Pro Plus software and quantified as IOD, normalized to the corresponding β-actin band and expressed relative to the control group; all experiments were performed in triplicate.

### 2.9. Statistical Analysis

Statistical analyses were performed using SPSS software (version 23.0; SPSS Inc., Chicago, IL, USA). Proteomics, qRT-PCR, WB, IHC, and IF analyses were performed using biological replicates derived from three animals per group. For qRT-PCR and densitometric analyses, technical replicates were performed in triplicate. All graphs were generated using Prism 8.0 (GraphPad Inc., San Diego, CA, USA) and Illustrator 2024 (Adobe Software Inc., San Jose, CA, USA). After analysis of variance, pairwise comparisons were performed using least significant difference tests to control for type I error.

## 3. Results

### 3.1. Pathological Observations in Morphology and Ultrastructure of Dairy Cow MG

**Figure 1:**
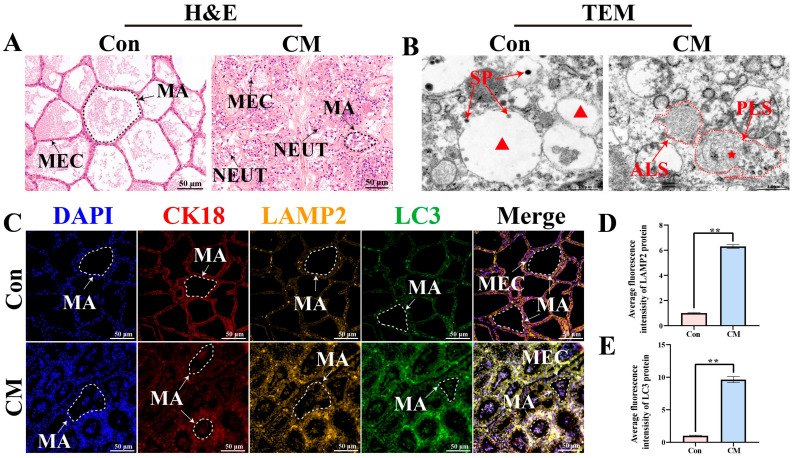
Histological and ultrastructural observations of bovine MG tissues: (**A**) Morphological structure of mammary acinus in the Con and CM groups. (**B**) Ultrastructural changes in MG tissues observed using TEM. (**C**) Colocalization analysis of CK18, LAMP2, and LC3 in MG tissues. (**D**,**E**) Quantitative analysis of the IOD values of LC3 and LAMP2 signals using Image-Pro Plus software. ALS, autolysosomes; MA, mammary acinus; NEUT, neutrophils; PLS, phagolysosomes; SP, secretory particles. The red star indicates primary lysosomes, and the red triangle indicates cytoplasmic vesicles. Scale bars: 50 μm (200×) and 1 μm (15.0k×). Negative controls for LAMP2 and LC3 are shown in Supplementary Figure S1. ** indicates *p* < 0.01.

H&E staining revealed that in the Con group, mammary acini were well filled, and the epithelial cells were arranged in an orderly manner, exhibiting cuboidal or columnar morphology with round or oval nuclei. In contrast, the CM group displayed a disorganized tissue structure, epithelial cell desquamation, shrinkage and deformation of the alveolar structures, and infiltration of neutrophils into the alveolar lumen, which were identified based on their classical H&E features, including multilobed nuclei and granular cytoplasm. (Figure 1A). TEM showed autolysosomes (ALS) and phagolysosomes (PLS) in MG tissues of the CM group (Figure 1B). IF staining demonstrated that both LAMP2 (a lysosomal marker) and LC3 (an autophagosome marker) exhibited fluorescent signals in MG tissues of the Con and CM groups (Figure 1C). The results indicated that CK18, LAMP2, and LC3 were colocalized in the cytoplasm of MECs. Compared with the Con group, the CM group exhibited stronger fluorescence intensities for LAMP2 and LC3 (Figure 1D,E). These findings suggest that autophagy-related structures and markers are associated with CM in dairy cows.

### 3.2. Screening of BPs Related to External Stimuli and Identification of Core DEPs

**Figure 2:**
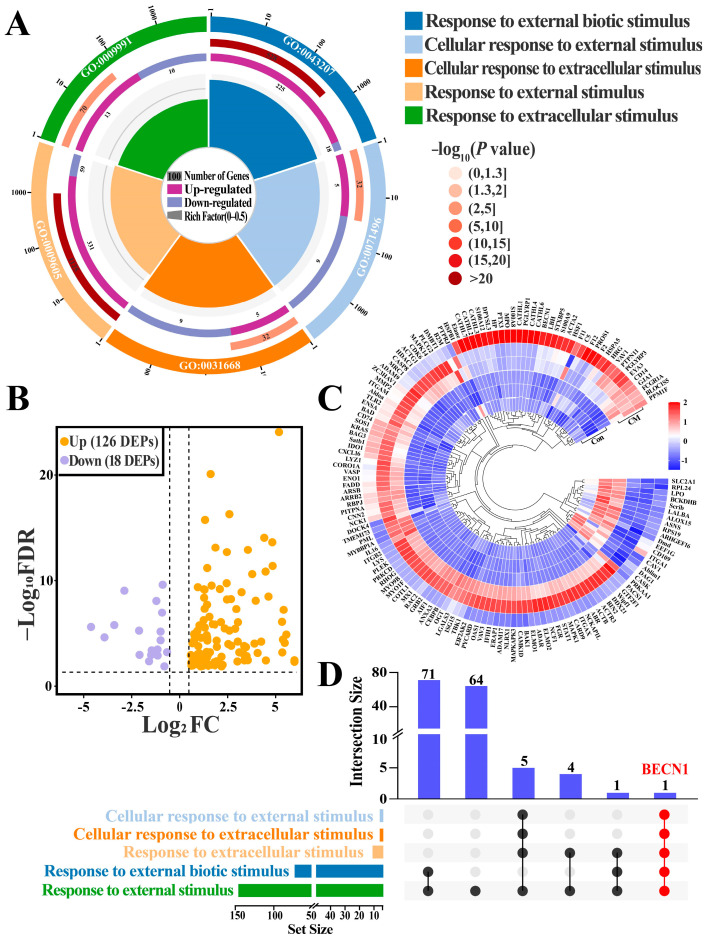
Identification of BPs and candidate DEPs associated with responses to external stimuli: (**A**) BPs related to responses to external stimuli. (**B**) Volcano plot showing the distribution of 144 candidate DEPs. (**C**) Heatmap of the expression profiles of 144 candidate DEPs. (**D**) UpSet diagram illustrating all DEPs shared among the five BPs.

GO enrichment analysis identified five BPs related to responses to external stimuli. (*p* < 0.05; Table S2), including cellular responses to external stimuli, cellular responses to extracellular stimuli, responses to external biotic stimuli, responses to external stimuli and responses to extracellular stimuli (Figure 2A). The five BPs collectively contained 144 DEPs. Compared with the Con group, 18 DEPs were downregulated and 126 DEPs were upregulated in the CM group (Figure 2B,C). Venn analysis further demonstrated that BECN1 was commonly involved in all five BPs (Figure 2D). These results suggested that changes in BECN1 expression are closely correlated with CM occurrence and progression in dairy cows, especially under conditions involving external stimuli.

### 3.3. Enrichment Analysis of KEGG Pathways Involving BECN1

**Figure 3:**
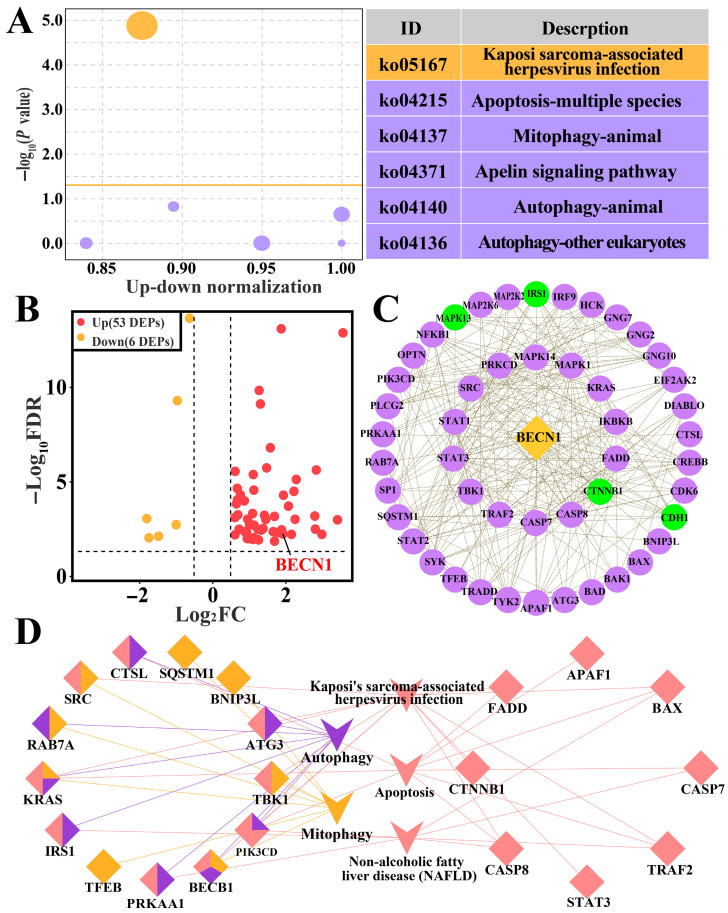
Screening of KEGG pathways and candidate DEPs associated with BECN1: (**A**) Bubble chart showing KEGG pathways associated with BECN1. (**B**) Volcano plot displaying 59 candidate DEPs. (**C**) PPI network of the 59 DEPs; green nodes represent downregulated proteins and purple nodes represent upregulated proteins. (**D**) ClueGO analysis of key candidate DEPs.

KEGG enrichment analysis revealed that BECN1 was involved in six pathways (Table S3), including Kaposi’s sarcoma-associated herpesvirus infection, apoptosis, the apelin signaling pathway, mitophagy, autophagy-animal, and autophagy-other eukaryotes (Figure 3A). A total of 59 DEPs were enriched in these six pathways, among which 53 DEPs were upregulated, and six DEPs were downregulated (Figure 3B). According to the PPI network 51 DEPs, including 47 upregulated and four downregulated proteins, interacted with BECN1 (Figure 3C). According to ClueGO analysis, BECN1 interacted with 12 DEPs, including Ras-related protein Rab-7a (RAB7A), Kirsten rat sarcoma viral oncogene homolog (KRAS), sequestosome 1 (SQSTM1), and autophagy-related protein 3 (ATG3). These proteins were involved in autophagy-related signaling pathways (Figure 3D). Collectively, these findings suggest that BECN1 is closely associated with bovine mastitis and may be involved in autophagy-related pathways.

### 3.4. Integrated Analysis of Biological Processes and KEGG Pathways

**Figure 4:**
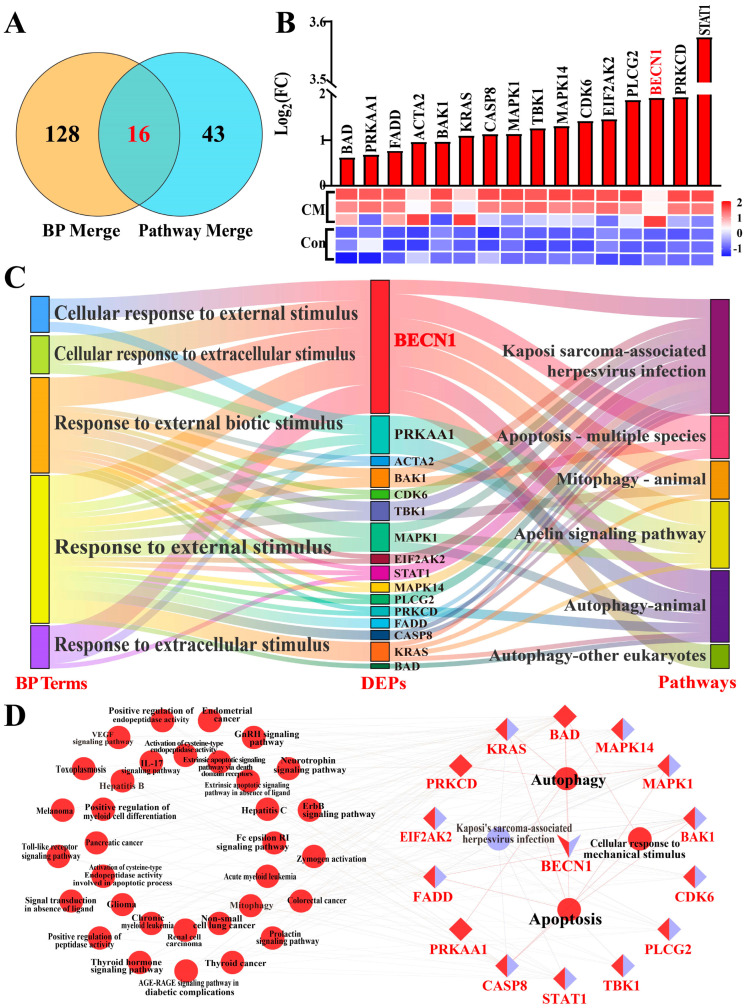
Integrated analysis of BPs and KEGG pathways identifying core DEPs associated with external stimuli and autophagy: (**A**) Venn diagram showing candidate DEPs shared among the five BPs and six KEGG pathways. (**B**) Heatmap of 16 key DEPs showing their expression patterns. (**C**) Sankey diagram illustrating the associations of 16 DEPs with five BPs and six KEGG pathways. (**D**) PPI network of the 16 key DEPs constructed using ClueGO.

The BPs associated with external stimuli and KEGG pathways were subjected to integrated analysis (Table S4). Venn analysis revealed that 16 DEPs were shared between the BPs and KEGG pathways (Figure 4A). All 16 DEPs were upregulated in the CM group, showing significant differences compared with the Con group (*p* < 0.01; Figure 4B). BECN1, as a core DEP, exhibited the strongest association with five BPs and six KEGG pathways (Figure 4C). PPI analysis revealed that BECN1, as a central protein, participated in multiple pathways related to responses to external stimuli, such as IL-17–mediated inflammatory response, gonadotropin-releasing hormone–mediated hormonal regulation, and Kaposi’s sarcoma–associated herpesvirus infection. Additionally, BECN1 interacted with several key proteins, including AMPK, Bcl-2-associated death promoter and TANK-binding kinase 1, which are jointly involved in autophagy regulatory pathways (Figure 4D). These findings suggest that BECN1 is closely associated with inflammatory responses in MG tissues during CM in dairy cows, potentially in relation to autophagy and external stimulus–related biological processes.

### 3.5. Expression Localization and Expression Patterns of BECN1 in Bovine MG Tissues

**Figure 5:**
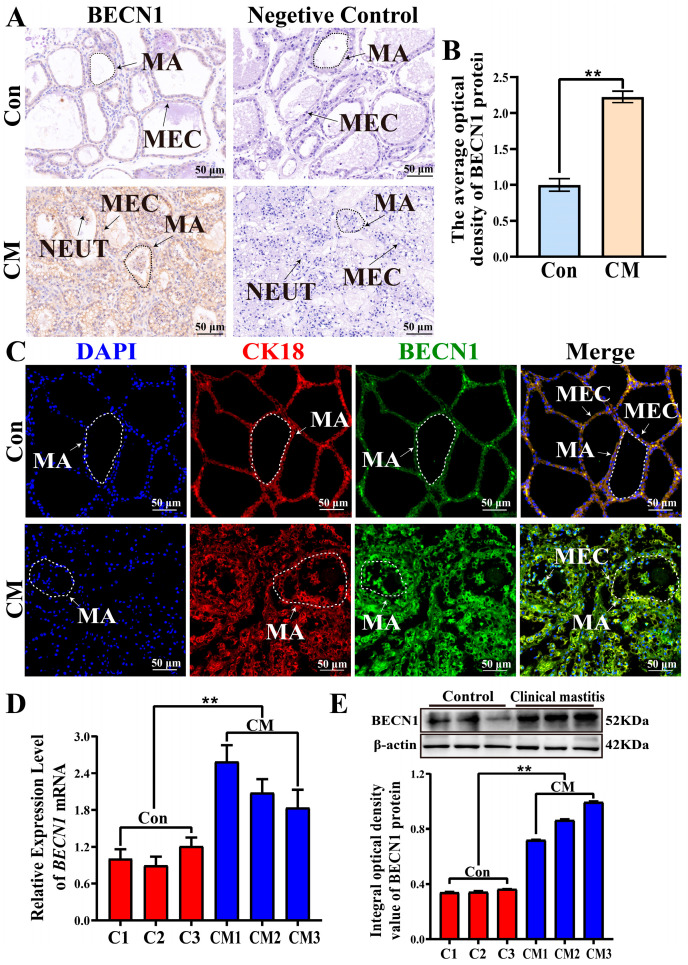
Expression localization and expression patterns of BECN1 in bovine MG tissues: (**A**) Distribution of BECN1 in MG tissues. (**B**) Quantitative analysis of the IOD values. (**C**) Colocalization analysis of CK18 and BECN1 in MG tissues. (**D**) Relative mRNA expression level of *BECN1* (**E**). Relative protein expression level of BECN1. Scale bars: 50 μm (200×). Negative control for BECN1 is shown in Supplementary Figure S2. Complete WBs are shown in Supplementary Figures S3 and S4. ** represents *p* < 0.01.

IHC analysis revealed that the BECN1 protein was localized in the cytoplasm of MECs. The staining intensity of BECN1 in the CM group was stronger than that in the Con group, whereas no positive staining was observed in the negative control (Figure 5A). IOD analysis showed that the expression level of BECN1 was significantly higher in the CM group than in the Con group (*p* < 0.01, Figure 5B). IF staining demonstrated that CK18 and BECN1 were colocalized in the cytoplasm of MECs, and the fluorescence intensity of BECN1 was stronger in the CM group than in the Con group (Figure 5C). qRT-PCR analysis showed that the mRNA levels of *BECN1* in the CM group were significantly higher than those in the Con group (*p* < 0.01, Figure 5D). Similarly, WB analysis revealed that BECN1 protein was expressed in both the Con and CM groups, and its abundance was significantly higher in the CM group (*p* < 0.01, Figure 5E). Collectively, these results suggest that upregulation of *BECN1* mRNA and protein expression was positively correlated with CM status. The colocalization of BECN1 with CK18 in the cytoplasm of MECs further confirmed that BECN1 expression was closely associated with its functional role in MECs. These findings suggest that increased BECN1 expression correlates with CM occurrence and progression and may be associated with autophagy-related pathways in MECs. These findings suggest that elevated BECN1 expression may contribute to the occurrence and progression of CM by regulating autophagy in MECs.

## 4. Discussion

Inflammatory responses in dairy cow MG can be triggered by various external factors, including environmental pollution, bacterial infections, improper milking practices, and imbalanced supplemental nutrition [27]. These external stimuli trigger mastitis by disrupting the blood–milk barrier, damaging MG tissues, and eliciting abnormal immune responses [28]. Abnormal external stimuli not only suppress the activity of metabolic and antioxidant enzymes in the MG [29,30], but also promote the infiltration of immune cells [31] and epithelial cell injury, thereby aggravating inflammation and facilitating the progression of CM.

The blood–milk barrier, formed by tight junctions of MECs, serves as a critical defense line in the MG. The disruption of this barrier allows pathogenic microorganisms to invade and activate the inflammatory cascade responses [32]. H&E staining revealed epithelial cell shedding and inflammatory cell infiltration in the CM group, consistent with the pathological features of blood–milk barrier disruption caused by external stimuli, such as bacterial infection and mechanical injury. These results suggest that once the barrier integrity is compromised, exogenous entities, including pathogens, can more readily penetrate the MG tissue and trigger inflammatory cascades. The host initiates an immune response through the activation of immune cells, phagocytic activity, and other defense mechanisms to counteract or eliminate external stimuli. TEM revealed the presence of ALS and PLS in the CM group, suggesting that autophagy occurs in MECs and may contribute to the elimination of invading pathogens and damaged organelles; however, the persistent destruction of mammary alveolar structures indicates that autophagy was insufficient to fully compensate for the tissue damage caused by continuous external stimulation [33]. Furthermore, the colocalization of LAMP2 and LC3 confirmed the formation of autolysosomes [34]. Autolysosomes degrade damaged organelles and proteins to suppress inflammation and maintain intracellular homeostasis [35]. Collectively, these findings indicate that autophagy plays an important role in the occurrence and development of bovine CM. However, the precise molecular mechanisms underlying this regulation remain unclear.

Based on previous DIA proteomic and bioinformatics analyses, BECN1 was identified as a key target associated with the host response to external stimuli and inflammatory reactions in MG tissues. Previous studies demonstrated that in a lipopolysaccharide-induced yak endometrial epithelial cell model, silencing BECN1 inhibited nuclear transcription factor-κB (NF-κB) activation and reduced TNF-α and IL-1β secretion, suggesting that BECN1 downregulation suppresses inflammation [36]. In an Alzheimer’s disease model, reduced BECN1 expression impaired the autophagic capacity of microglial cells, resulting in enhanced activation of the NOD-, LRR- and pyrin domain-containing protein 3 (NLRP3) inflammasome and a significant increase in IL-1β and IL-18 secretion [37]. These findings indicate a close association between BECN1 expression and inflammatory responses. KEGG analysis revealed that BECN1 participates in several autophagy-related signaling pathways, including mitophagy, animal autophagy, and eukaryotic autophagy, suggesting that BECN1, as a key DEP, may be associated with bovine CM in relation to autophagy-associated mechanisms. Accumulating evidence suggests that BECN1 is a central regulator of autophagy that facilitates autophagosome formation and initiates autophagy to alleviate cellular injury [38]. Moreover, BECN1 modulates the NF-κB pathway through its role in autophagy, thereby influencing the inflammatory responses [39]. In addition, BECN1 can activate the NLRP3 inflammasome through selective autophagy, controlling IL-1β and IL-18 production and mediating neural and tissue inflammation [37]. IHC and IF analyses showed that BECN1 was predominantly localized in the cytoplasm of MECs in the CM group, indicating that MECs activate the autophagic pathway in response to inflammatory stress to regulate mammary inflammation. As a core component of the autophagy initiation complex (BECN1–VPS34–ATG14L), BECN1 participates in autophagosome formation and membrane elongation [38]; cytoplasmic localization is consistent with its functional characteristics in autophagy. qRT-PCR and WB analyses showed that *BECN1* expression was significantly upregulated at both mRNA and protein levels. Upregulated BECN1 expression generally indicates enhanced autophagic activity, which contributes to the clearance of damaged organelles and oxidative products, and maintains intracellular homeostasis [40]. However, under persistent infectious stimulation, excessive or prolonged autophagy may fail to completely compensate for inflammatory injury [33], suggesting that maintaining a balance between autophagy and inflammation is crucial during the pathological progression of mastitis.

**Figure 6:**
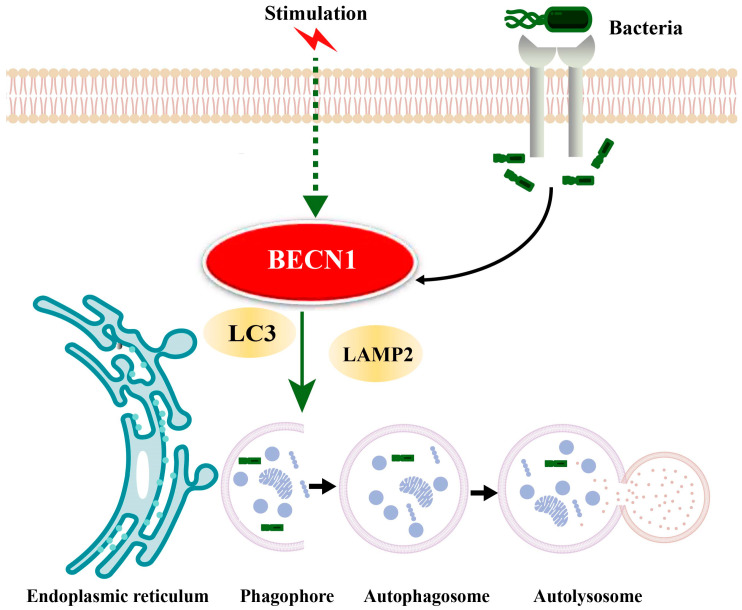
Proposed molecular mechanism by which external stimuli regulate BECN1-mediated autophagy in CM of dairy cows.

In summary, we propose a potential mechanism for BECN1 function in bovine CM based on the results of this study and KEGG pathway analysis (Figure 6). Upon abnormal exogenous stimulation or pathogenic invasion, BECN1 initiates autophagy, LC3 participates in autophagosome formation, and LAMP2 mediates the fusion of autophagosomes with lysosomes to form autolysosomes, which subsequently degrade the contents encapsulated within autophagosomes [41].

This study has several limitations. First, all CM cases analyzed were caused by *Staphylococcus aureus*, potentially limiting the generalizability of our findings to mastitis induced by other pathogens. Therefore, the present results should be considered exploratory and hypothesis-generating. Future studies that integrate in vitro mammary epithelial cell models with in vivo animal experiments are warranted to validate the functional role of BECN1 in CM. Moreover, this study primarily focused on the initiation of autophagy; therefore, a comprehensive evaluation of autophagic flux and its interaction with inflammatory responses is required. Given that BECN1 may interact with other autophagy-related molecules, including ATG5, ATG12, and LC3 [42], further investigation of these molecular networks will be essential to fully elucidate the role of autophagy in CM. Future studies should explore the interactions among these molecules to elucidate their coordinated regulatory networks.

## 5. Conclusions

This study demonstrates that CM in dairy cows is accompanied by pronounced inflammatory injury and the involvement of autophagy-related processes in MG tissues. Through integrative proteomic, bioinformatic, bioinformatics, histological, and molecular analyses, BECN1 was identified as a central biomolecule that is consistently associated with biological processes responsive to external stimuli and autophagy-related pathways. This study proposes a potential mechanism by which BECN1 may be involved in the pathological progression of clinical mastitis induced by external stimuli via autophagy-related pathways, thereby providing new insights and a theoretical basis for future prevention and treatment strategies for bovine CM. Future studies should explore the dynamic roles of BECN1 in autophagy-related processes using both in vitro and in vivo models.

## Data Availability

The data that support the findings of this study are available from the corresponding author upon reasonable request.

## Supplementary Materials

The following supporting information can be downloaded at https://www.mdpi.com/article/10.3390/biom16010133/s1. Table S1: Primer sequences. Table S2: Biological processes analysis. Table S3: Pathways analysis; Table S4: BPs and pathways analysis. Table S5: The used antibodies and dilution ratio in the present study. Figures S1 and S2: Negative controls for LAMP2, LC3 and BECN1. Figures S3 and S4: Whole Western blot of BECN1 (52 kDa), and β-actin (42 kDa).

## Abbreviations

The following abbreviations are used in this manuscript:

AMPK	AMP-activated protein kinase
ALS	Autolysosomes
ATG12	Autophagy-related protein 12
ATG3	Autophagy-related protein 3
ATG5	Autophagy-related protein 5
BAD	Bcl-2-associated death promoter
BECN1	Beclin 1
BPs	Biological processes
CaMKKβ	Calmodulin-dependent protein kinase II β
CM	Clinical mastitis
Con	Control
DIA	Data-independent acquisition
DEPs	Differentially expressed proteins
ER	Endoplasmic reticulum
ECL	Enhanced chemiluminescence
GO	Gene Ontology
GnRH	Gonadotropin-releasing hormone
HtrA2	High-temperature requirement protein A2
H_2_O_2_	Hydrogen peroxide
IL-18	Interleukin-18
IL-1β	Interleukin-1β
IL-6	Interleukin-6
KRAS	Kirsten rat sarcoma viral oncogene homolog
KEGG	Kyoto Encyclopedia of Genes and Genomes
LPS	Lipopolysaccharide
LAMP2	Lysosomal-associated membrane protein 2
LMT	Lanzhou mastitis test
MA	Mammary acinus
MECs	Mammary epithelial cells
MG	Mammary gland
LC3	Microtubule-associated protein 1 light chain 3
NEUT	Neutrophils
NLRP3	NOD-, LRR- and pyrin domain-containing protein 3
NF-κB	Nuclear transcription factor-κB
ANOVA	One-way analysis of variance
OPA1	Optic atrophy 1
PLS	Phagolysosomes
PMSF	Phenylmethylsulfonyl fluoride
PBS	Phosphate-buffered saline
PVDF	Polyvinylidene fluoride
PIC	Protease inhibitor cocktail
PPI	Protein–protein interaction
qRT-PCR	Quantitative real-time PCR
RAB7A	Ras-related protein Rab-7a
ROS	Reactive oxygen species
SP	Secretory particles
SQSTM1	Sequestosome 1
LSD-t	Significant difference test
SCC	Somatic cell count
SD	Standard deviation
SABC	Streptavidin–biotin complex
TBK1	TANK-binding kinase 1
TNF-α	Tumor necrosis factor-α
XIAP	X-linked inhibitor of apoptosis protein

## References

[1] Jiang L., Li Q., Liao H., Liu H., Wang Z. (2025). Enhancing Agricultural Productivity in Dairy Cow Mastitis Management: Innovations in Non-Antibiotic Treatment Technologies. Vet. Sci..

[2] Cheng Z., Buggiotti L., Salavati M., Marchitelli C., Palma-Vera S., Wylie A., Takeda H., Tang L., Crowe M.A., Wathes D.C. (2021). Global transcriptomic profiles of circulating leucocytes in early lactation cows with clinical or subclinical mastitis. Mol. Biol. Rep..

[3] Bar D., Gröhn Y., Bennett G., González R., Hertl J., Schulte H., Tauer L., Welcome F., Schukken Y. (2008). Effects of repeated episodes of generic clinical mastitis on mortality and culling in dairy cows. J. Dairy Sci..

[4] Bannerman D. (2009). Pathogen-dependent induction of cytokines and other soluble inflammatory mediators during intramammary infection of dairy cows. J. Anim. Sci..

[5] Wellnitz O., Bruckmaier R.M. (2012). The innate immune response of the bovine mammary gland to bacterial infection. Vet. J..

[6] Rainard P., Gilbert F.B., Germon P. (2022). Immune defenses of the mammary gland epithelium of dairy ruminants. Front. Immunol..

[7] Hoekstra J., Rutten V.P., Lam T.J., Van Kessel K.P., Spaninks M.P., Stegeman J.A., Benedictus L., Koop G. (2019). Activation of a bovine mammary epithelial cell line by ruminant-associated Staphylococcus aureus is lineage dependent. Microorganisms.

[8] Chen R., Zou J., Chen J., Zhong X., Kang R., Tang D. (2025). Pattern recognition receptors: Function, regulation and therapeutic potential. Signal Transduct. Target. Ther..

[9] Herve L., Quesnel H., Lollivier V., Portanguen J., Bruckmaier R., Boutinaud M. (2017). Mammary epithelium disruption and mammary epithelial cell exfoliation during milking in dairy cows. J. Dairy Sci..

[10] Suzuki Y., Imai Y., Nakayama H., Takahashi K., Takio K., Takahashi R. (2001). A serine protease, HtrA2, is released from the mitochondria and interacts with XIAP, inducing cell death. Mol. Cell.

[11] Yan L., Yang Y., Ma X., Wei L., Wan X., Zhang Z., Ding J., Peng J., Liu G., Gou H. (2022). Effect of two different drug-resistant Staphylococcus aureus strains on the physiological properties of MAC-T cells and their transcriptome analysis. Front. Vet. Sci..

[12] Cao S.S., Kaufman R.J. (2014). Endoplasmic reticulum stress and oxidative stress in cell fate decision and human disease. Antioxid. Redox Signal..

[13] Lee J., Giordano S., Zhang J. (2012). Autophagy, mitochondria and oxidative stress: Cross-talk and redox signalling. Biochem. J..

[14] Yang Y., Li M., Wang J., Zhang H., Chang R., Zhao B., Aernouts B., Huang Q., Xu C. (2025). Carvacrol alleviates endoplasmic reticulum stress and inflammation induced by lipopolysaccharide by enhancing endoplasmic reticulum autophagy in dairy mammary epithelial cells. J. Dairy Sci..

[15] Zhao Z., Hou Z., Chai J., Li C., Liu T., Li J., Zhang S., Zhang L., Ma Y. (2025). Mechanisms of host-bacterial interactions during Escherichia coli or Staphylococcus aureus infection of mammary epithelial cells. Front. Vet. Sci..

[16] Khan S., Yang J., Cobo E.R., Wang Y., Xu M., Wang T., Shi Y., Liu G., Han B. (2023). Streptococcus uberis induced expressions of pro-inflammatory IL-6, TNF-α, and IFN-γ in bovine mammary epithelial cells associated with inhibited autophagy and autophagy flux formation. Microb. Pathog..

[17] Pang Y., Wu L., Tang C., Wang H., Wei Y. (2022). Autophagy-inflammation interplay during infection: Balancing pathogen clearance and host inflammation. Front. Pharmacol..

[18] Deretic V. (2021). Autophagy in inflammation, infection, and immunometabolism. Immunity.

[19] Meng M., Li X., Wang Z., Huo R., Ma N., Chang G., Shen X. (2023). A high-concentrate diet induces inflammatory injury via regulating Ca2+/CaMKKβ-mediated autophagy in mammary gland tissue of dairy cows. Front. Immunol..

[20] Bai X., Wang X., Lin T., Dong W., Gao Y., Ji P., Zhang Y., Zhao X., Zhang Q. (2022). Toll-like receptor 2 is associated with the immune response, apoptosis, and angiogenesis in the mammary glands of dairy cows with clinical mastitis. Int. J. Mol. Sci..

[21] Zhang Q., Wang Q., Gong J., Du J., Zhang Y., Zhao X. (2018). Yak igf2 promotes fibroblast proliferation via suppression of igf1r and pi3kcg expression. Genes.

[22] Sadat A., Farag A.M., Elhanafi D., Awad A., Elmahallawy E.K., Alsowayeh N., El-Khadragy M.F., Elshopakey G.E. (2023). Immunological and oxidative biomarkers in bovine serum from healthy, clinical, and sub-clinical mastitis caused by Escherichia coli and Staphylococcus aureus infection. Animals.

[23] He W., Sun Z., Tong G., Zeng L., He W., Chen X., Zhen C., Chen P., Tan N., He P. (2024). FUNDC1 alleviates doxorubicin-induced cardiotoxicity by restoring mitochondrial-endoplasmic reticulum contacts and blocked autophagic flux. Theranostics.

[24] Zhang B., Lin T., Bai X., An X., Dai L., Shi J., Zhang Y., Zhao X., Zhang Q. (2022). Sulfur Amino Acid Metabolism and the Role of Endogenous Cystathionine-γ-lyase/H2S in Holstein Cows with Clinical Mastitis. Animals.

[25] Livak K.J., Schmittgen T.D. (2001). Analysis of relative gene expression data using real-time quantitative PCR and the 2−ΔΔCT method. Methods.

[26] Zhang Q., Bai X., Shi J., Wang X., Zhang B., Dai L., Lin T., Gao Y., Zhang Y., Zhao X. (2022). DIA proteomics identified the potential targets associated with angiogenesis in the mammary glands of dairy cows with hemorrhagic mastitis. Front. Vet. Sci..

[27] Ashraf A., Imran M. (2020). Causes, types, etiological agents, prevalence, diagnosis, treatment, prevention, effects on human health and future aspects of bovine mastitis. Anim. Health Res. Rev..

[28] Ezzat Alnakip M., Quintela-Baluja M., Böhme K., Fernández-No I., Caamaño-Antelo S., Calo-Mata P., Barros-Velázquez J. (2014). The immunology of mammary gland of dairy ruminants between healthy and inflammatory conditions. J. Vet. Med..

[29] Xu T., Liu R., Zhu H., Zhou Y., Pei T., Yang Z. (2022). The inhibition of LPS-induced oxidative stress and inflammatory responses is associated with the protective effect of (-)-Epigallocatechin-3-Gallate on bovine hepatocytes and murine liver. Antioxidants.

[30] Wang J., Zhang X., He X., Yang B., Wang H., Shan X., Li C., Sun D., Wu R. (2018). LPS-induced reduction of triglyceride synthesis and secretion in dairy cow mammary epithelial cells via decreased SREBP1 expression and activity. J. Dairy Res..

[31] Choudhary R.K., Olszanski L., McFadden T.B., Lalonde C., Spitzer A., Shangraw E.M., Rodrigues R.O., Zhao F.-Q. (2024). Systemic and local responses of cytokines and tissue histology following intramammary lipopolysaccharide challenge in dairy cows. J. Dairy Sci..

[32] Wellnitz O., Bruckmaier R. (2021). The role of the blood-milk barrier and its manipulation for the efficacy of the mammary immune response and milk production. J. Dairy Sci..

[33] Liu Y., Deng Z., Xu S., Liu G., Lin Y., Khan S., Gao J., Qu W., Kastelic J.P., Han B. (2021). Mycoplasma bovis subverts autophagy to promote intracellular replication in bovine mammary epithelial cells cultured in vitro. Vet. Res..

[34] Eskelinen E.-L., Illert A.L., Tanaka Y., Schwarzmann G., Blanz J., Von Figura K., Saftig P. (2002). Role of LAMP-2 in lysosome biogenesis and autophagy. Mol. Biol. Cell.

[35] Mahapatra K.K., Mishra S.R., Behera B.P., Patil S., Gewirtz D.A., Bhutia S.K. (2021). The lysosome as an imperative regulator of autophagy and cell death. Cell. Mol. Life Sci..

[36] Ma W., Wang L., Pan Y., Wang M., Wang J., Feng M., Wang J., Zhang H., Zhang R., Jiao Z. (2025). Beclin1 regulates yak endometrial inflammation and TLR4/NF-κB signaling pathway through autophagy/non-autophagy function. Int. Immunopharmacol..

[37] Houtman J., Freitag K., Gimber N., Schmoranzer J., Heppner F.L., Jendrach M. (2019). Beclin1-driven autophagy modulates the inflammatory response of microglia via NLRP 3. EMBO J..

[38] Kang R., Zeh H., Lotze M., Tang D. (2011). The Beclin 1 network regulates autophagy and apoptosis. Cell Death Differ..

[39] Lv Y., Fang L., Ding P., Liu R. (2019). PI3K/Akt-Beclin1 signaling pathway positively regulates phagocytosis and negatively mediates NF-κB-dependent inflammation in Staphylococcus aureus-infected macrophages. Biochem. Biophys. Res. Commun..

[40] Wang X., Sun D., Hu Y., Xu X., Jiang W., Shang H., Cui D. (2019). The roles of oxidative stress and Beclin-1 in the autophagosome clearance impairment triggered by cardiac arrest. Free Radic. Biol. Med..

[41] Qi M., Geng H., Geng N., Cui Y., Qi C., Cheng G., Song K., Hu L., Liu Y., Liu J. (2022). Streptococcus agalactiae-induced autophagy of bovine mammary epithelial cell via PI3K/AKT/mTOR pathway. J. Dairy Res..

[42] Motyl T., Gajewska M., Zarzynska J., Sobolewska A., Gajkowska B. (2007). Regulation of autophagy in bovine mammary epithelial cells. Autophagy.

