# Supplementary figures and images for: Investigation of BECN1-Mediated Autophagy Mechanisms Triggered by External Stimuli in Clinical Mastitis of Dairy Cows
Source: Biomolecules. 2026 Jan 12;16(1):133. doi: 10.3390/biom16010133 (PMC12839052; doi:10.3390/biom16010133)

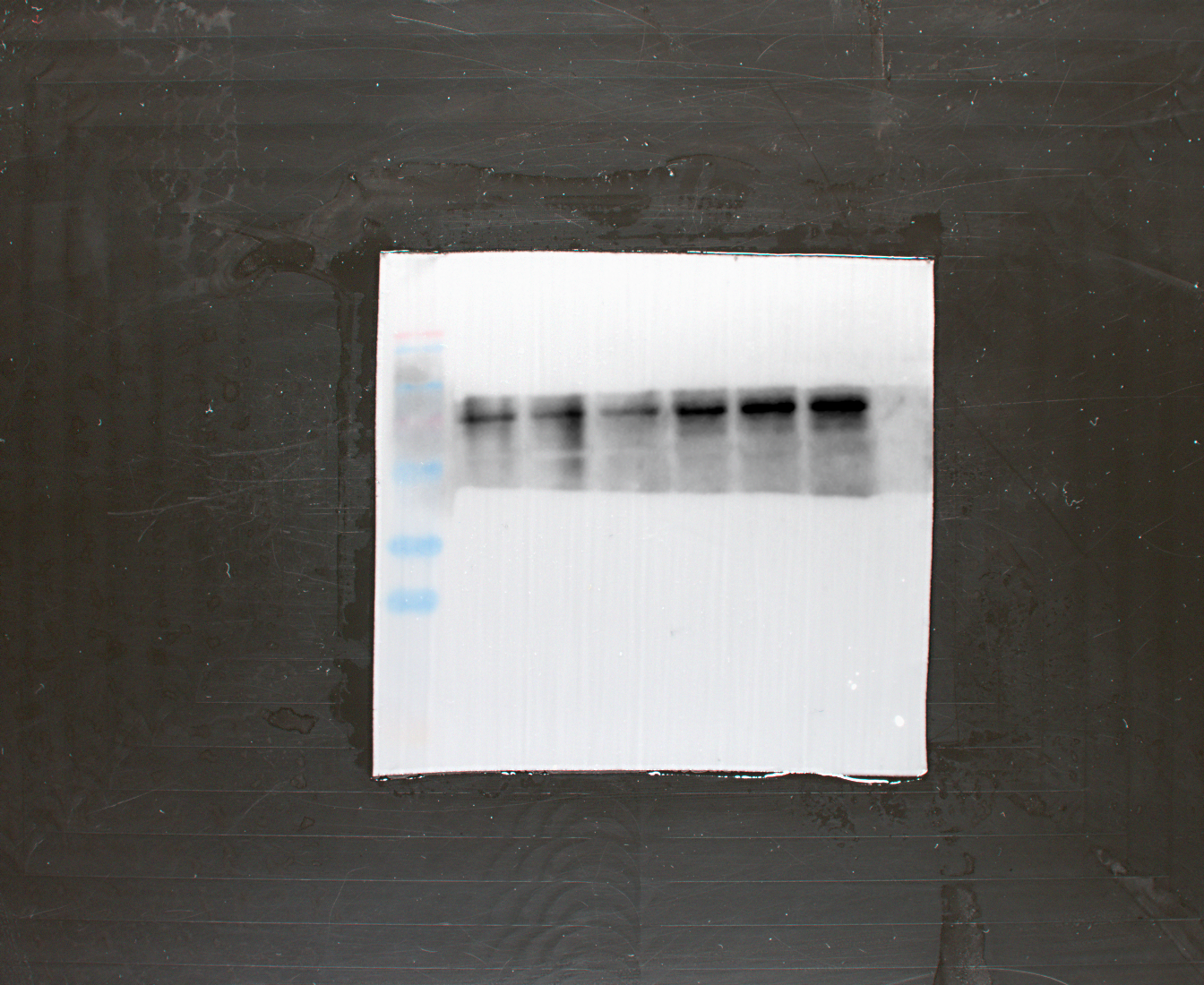

Supplement: Supplementary file 1 [file biomolecules-16-00133-s001.zip › Figure S3 (BECN1 52kDa).Tif]

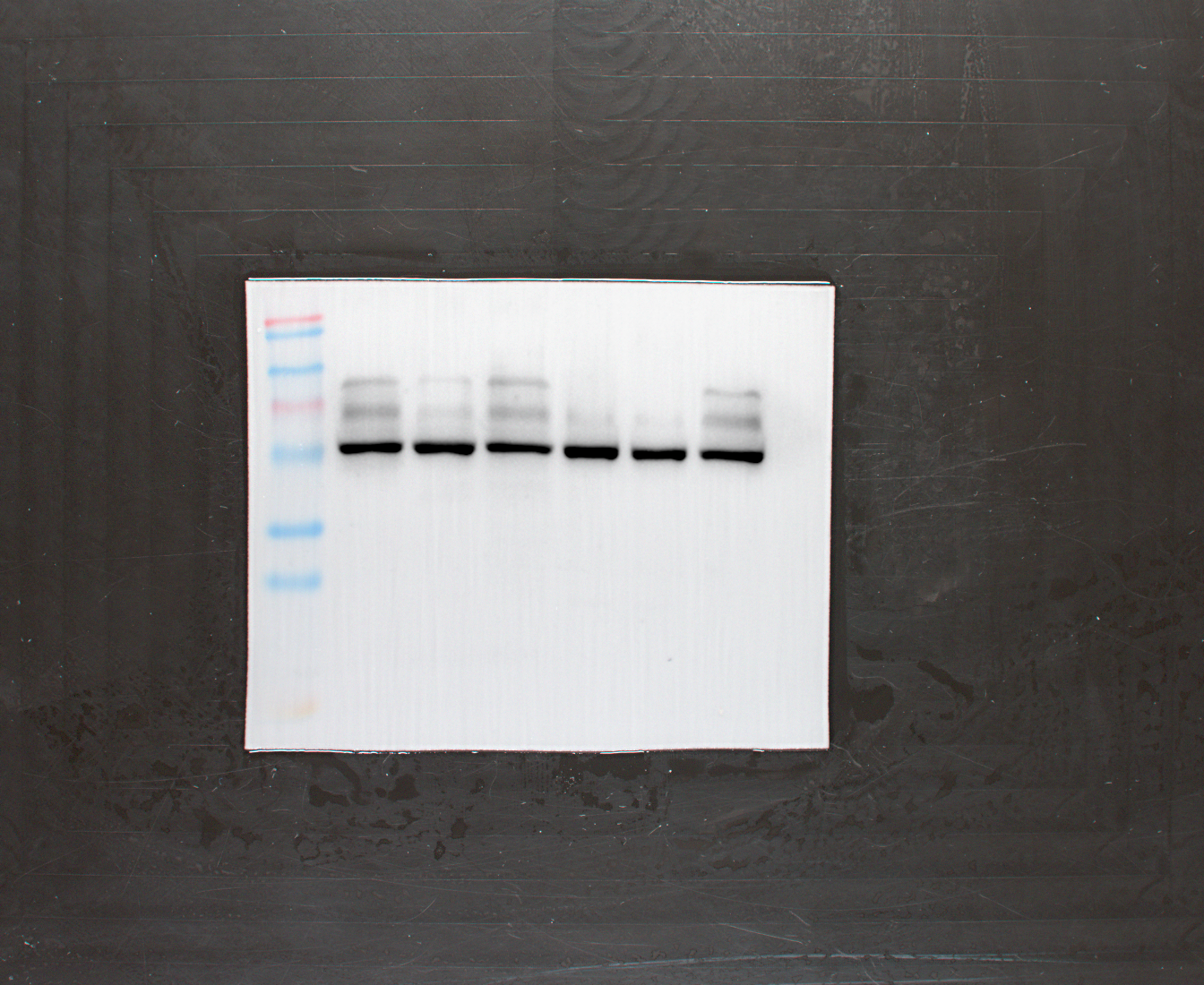

Supplement: Supplementary file 1 [file biomolecules-16-00133-s001.zip › Figure S4 (β-actin 42kDa).Tif]

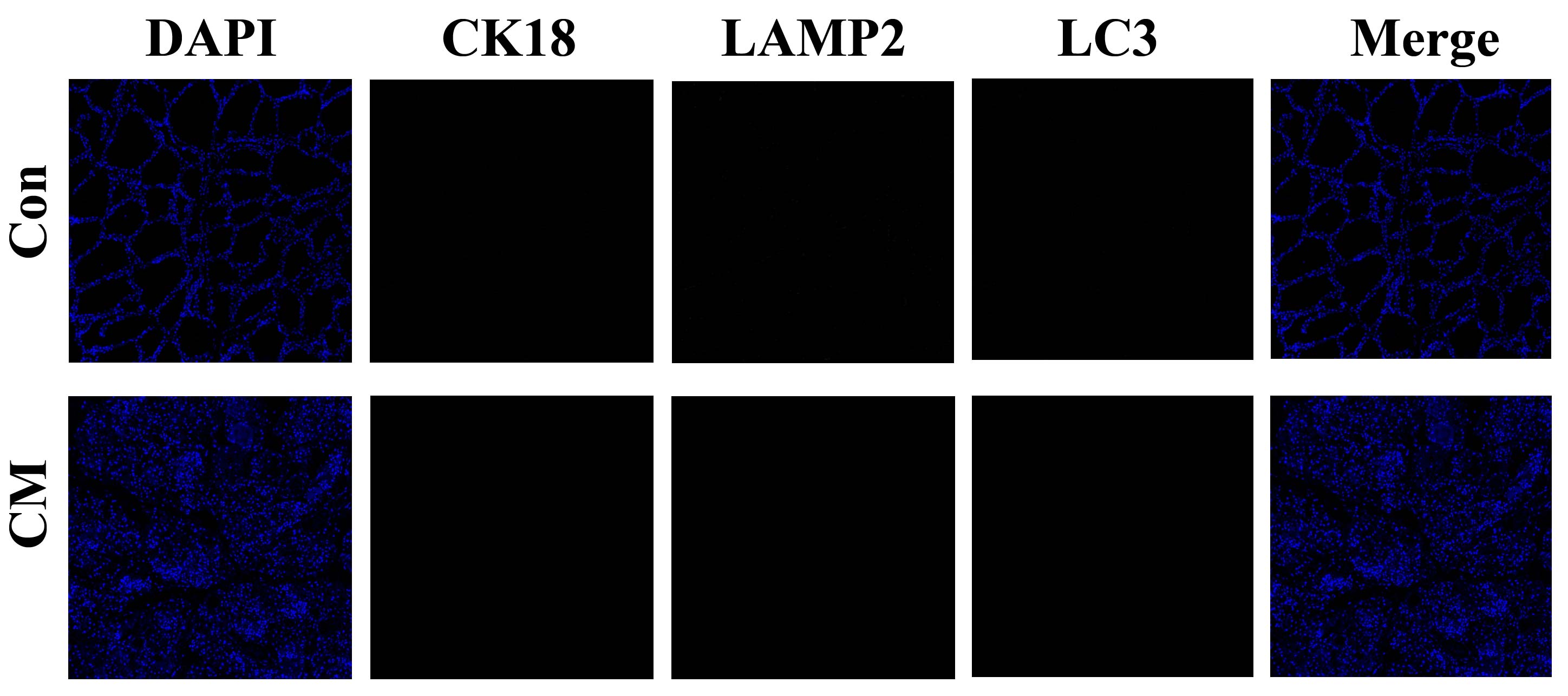

Supplement: Supplementary file 1 [file biomolecules-16-00133-s001.zip › Figure S1(LC3 LAMP2 Negative control).jpg]

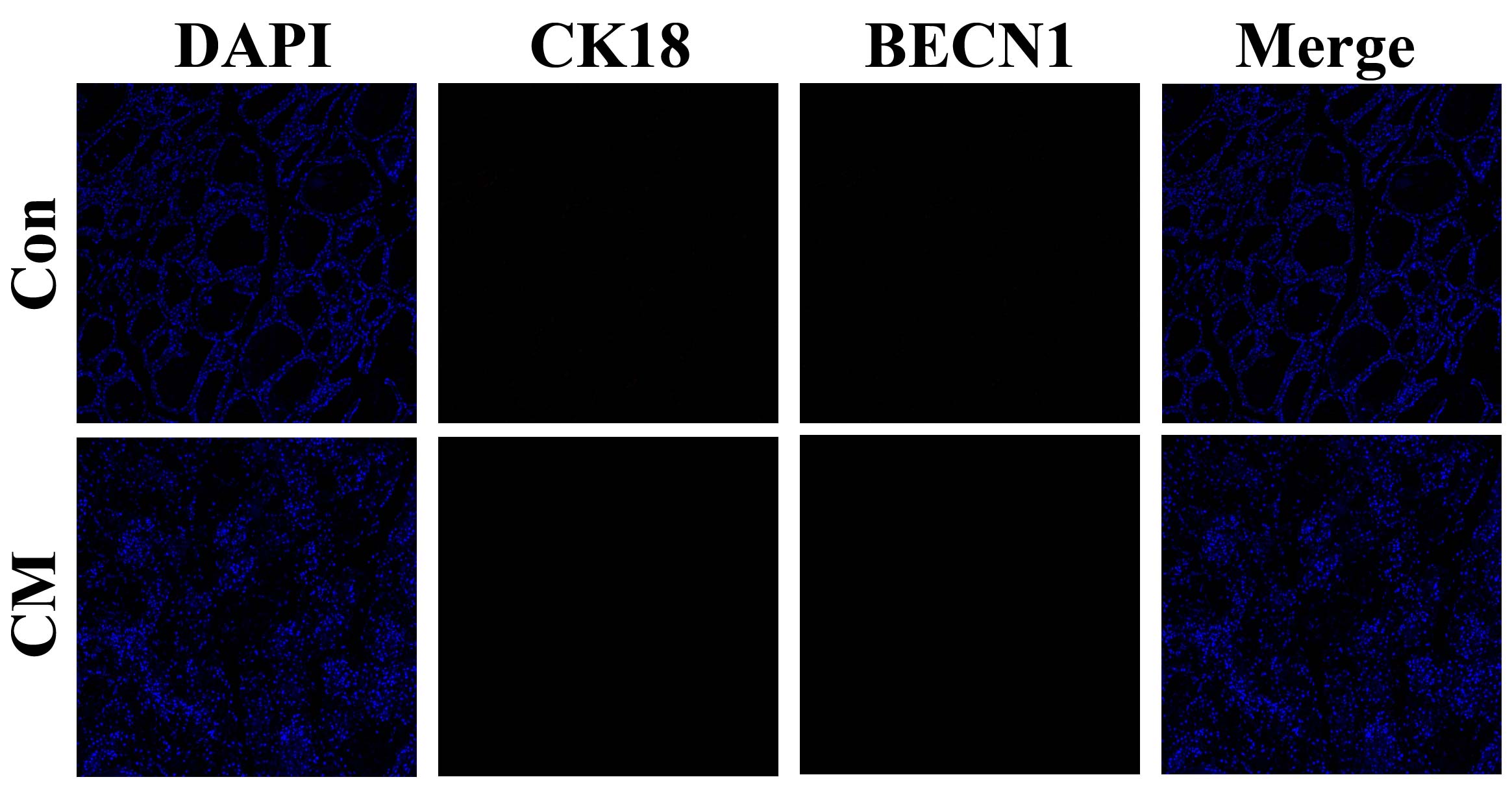

Supplement: Supplementary file 1 [file biomolecules-16-00133-s001.zip › Figure S2 (BECN1 Negative control).jpg]
